# Acute peripheral immune activation alters cytokine expression and glial activation in the early postnatal rat brain

**DOI:** 10.1186/s12974-019-1569-2

**Published:** 2019-10-31

**Authors:** Matthew Bruce, Karin M. Streifel, Casey A. Boosalis, Luke Heuer, Eduardo A. González, Shuyang Li, Danielle J. Harvey, Pamela J. Lein, Judy Van de Water

**Affiliations:** 1 MIND Institute, University of California, Davis School of Medicine, Sacramento, CA 95817 USA; 2 Department of Internal Medicine, Division of Rheumatology, Allergy and Clinical Immunology, University of California, UC Davis School of Medicine, UC Davis MIND Institute, 6512 Genome and Biomedical Sciences Facility 451 Health Sciences Drive, Davis, CA 95616-5270 USA; 3 Department of Molecular Bioscience, University of California, Davis School of Veterinary Medicine, Davis, CA 95616 USA; 4 Department of Biology, Regis University, Denver, CO 80221 USA; 5 Department of Public Health Sciences, University of California, Davis School of Medicine, Davis, CA 95616 USA

**Keywords:** Rat model, Cytokines, Microglia, Astrocytes, Sex differences, Peripheral immune challenge, Neuroinflammation, Neuroimmune

## Abstract

**Background:**

Neuroinflammation can modulate brain development; however, the influence of an acute peripheral immune challenge on neuroinflammatory responses in the early postnatal brain is not well characterized. To address this gap in knowledge, we evaluated the peripheral and central nervous system (CNS) immune responses to a mixed immune challenge in early postnatal rats of varying strains and sex.

**Methods:**

On postnatal day 10 (P10), male and female Lewis and Brown Norway rats were injected intramuscularly with either a mix of bacterial and viral components in adjuvant, adjuvant-only, or saline. Immune responses were evaluated at 2 and 5 days post-challenge. Cytokine and chemokine levels were evaluated in serum and in multiple brain regions using a Luminex multiplex assay. Multi-factor ANOVAs were used to compare analyte levels across treatment groups within strain, sex, and day of sample collection. Numbers and activation status of astrocytes and microglia were also analyzed in the cortex and hippocampus by quantifying immunoreactivity for GFAP, IBA-1, and CD68 in fixed brain slices. Immunohistochemical data were analyzed using a mixed-model regression analysis.

**Results:**

Acute peripheral immune challenge differentially altered cytokine and chemokine levels in the serum versus the brain. Within the brain, the cytokine and chemokine response varied between strains, sexes, and days post-challenge. Main findings included differences in T helper (Th) type cytokine responses in various brain regions, particularly the cortex, with respect to IL-4, IL-10, and IL-17 levels. Additionally, peripheral immune challenge altered GFAP and IBA-1 immunoreactivity in the brain in a strain- and sex-dependent manner.

**Conclusions:**

These findings indicate that genetic background and sex influence the CNS response to an acute peripheral immune challenge during early postnatal development. Additionally, these data reinforce that the developmental time point during which the challenge occurs has a distinct effect on the activation of CNS-resident cells.

**Electronic supplementary material:**

The online version of this article (10.1186/s12974-019-1569-2) contains supplementary material, which is available to authorized users.

## Background

An acute peripheral immune response can be widely systemic, affecting a variety of tissues and organ systems, although the tissue-specific response may vary greatly [1]. For example, peripheral immune stimulation has been shown to influence neuroinflammatory responses in the central nervous system (CNS) [2, 3]. Specific effects seen in the brain following peripheral immune challenge include global changes in expression of interferon response genes [4] as well as alterations in cell-specific transcriptional programming, particularly in microglia [5, 6]. These transcriptional alterations of neuroimmune signaling in early life are hypothesized to result in developmental priming, potentially leading to enduring consequences in response to later life exposures (reviewed in [7, 8]). Therefore, it is important to gain a deeper understanding of the relationship between peripheral inflammation and early postnatal CNS response, to evaluate the risk factors in early life as well as identify strategies to limit adverse effects.

Under physiologic conditions, immune signaling within the CNS is coordinated primarily by resident cells such as microglia, astrocytes, and mast cells due to tightly regulated infiltration of peripheral leukocytes into the brain parenchyma [9, 10]. When activated, these resident immune cells secrete a range of cytokines, chemokines, and other regulatory factors that drive neuroinflammatory responses and contribute to normal neurodevelopment and functional homeostasis [11, 12]. Integration of systemic immune signals by CNS-resident cells may occur via coordinated signaling through the autonomic nervous system and the hypothalamic-pituitary-adrenal (HPA) axis [13], trafficking and effector functions of immune cells within the meninges [14], and gut microbe-mediated mechanisms [15]. Each of these systems undergoes overlapping periods of development and refinement during the first few weeks following birth. Therefore, immune activation during these critical periods of development can have broad implications on neurodevelopment and neural function later in life.

In this study, we focused on understanding the relationship between the peripheral immune system and developing CNS by evaluating the respective immune responses to an acute peripheral, mixed immune challenge at an early postnatal time point. To do this, we challenged rats with either a mix of bacterial and viral components in adjuvant, adjuvant alone, or saline on postnatal day 10 (P10). The time point of P10 was chosen for exposure as it roughly translates to the first year of life in humans [16, 17] and represents an age of peak brain growth in rats [18]. We then evaluated the subsequent peripheral and CNS immune response 2 and 5 days later to compare early vs. late post-challenge immune responses. As a readout, we analyzed cytokine and chemokine levels in the serum as well as cortical, hippocampal, and cerebellar lysates. Additionally, we assessed the numbers and activation profiles of microglia and astrocytes within the cortex and hippocampus. Given the inherent heterogeneity in the immune response due to genetic background and sex [19, 20], an additional aim of the study was to compare the CNS and peripheral immune response following immune challenge in male and female Lewis and Brown Norway (BN) rats. These strains of rats were chosen as they exhibit immune response skewing, with Lewis rats skewing toward a T helper (Th) 1 cell (cellular, proinflammatory) response and BN rats skewing toward a Th2 (humoral, regulatory) response [21, 22]. While evidence exists suggesting sex-specific differences in CNS immune responsiveness during early postnatal development [23], the influence of genetic background on sex-specific immune responses is not as well documented. Therefore, a primary goal of this study was to identify potential differences in the region-specific CNS immune response in neonatal rats to a peripheral mixed immune challenge in the context of sex and genetic background.

## Methods

### Materials

The mixed acute peripheral immune challenge was comprised of hepatitis B (HepB) (Recombivax HB; Merck & Co., Whitehouse Station, New Jersey), diphtheria and tetanus toxoids and acellular pertussis (DTap) (DAPTACEL; Sanofi Pasteur, Swiftwater, Pennsylvania), Haemophilus influenza type b (Hib) (PedvaxHIB; Merck & Co., Inc., Whitehouse Station, New Jersey), pneumococcal conjugate (PCV) (Prevnar 13; Wyeth Pharmaceuticals Inc., Madison, New Jersey), and inactivated poliovirus (IPV) (IPOL; Sanofi Pasteur, Swiftwater, Pennsylvania) in adjuvant. To match the adjuvant pre-mixed with the above antigen preparations, a control adjuvant of 2% aluminum hydroxide gel (Alhydrogel) was obtained from InvivoGen (San Diego, California).

### Animals

All animals were housed in facilities fully accredited by AAALAC International, and all studies were performed with regard to the alleviation of pain and suffering under protocols approved by the University of California-Davis Institutional Animal Care and Use Committee. Lewis and Brown Norway (BN) timed-pregnant female rats (*n* = 6 per strain) were obtained from Charles River Laboratories (Portage, MI). Rats were individually housed on a 12:12-h light:dark cycle at 22 ± 2 °C with food and water available ad libitum. Lewis dams delivered litters of 10–12 pups on average, whereas BN dams delivered litters of 3–5 pups on average. On P7, littermates from each strain were sexed, randomly assigned to different experimental groups using a random number generator, and ear punched for identification purposes.

### Peripheral immune challenge

To trigger a full-spectrum innate and adaptive immune response, we utilized a peripheral immune challenge that included both bacterial and viral immune-stimulating agents. Offspring were injected intramuscularly (i.m.) on P10 with one of the following: acute peripheral immune challenge in adjuvant, adjuvant mixed with saline, or saline alone. The dose (0.105 μL/g) was determined based on the human dosing for this antigen mixture and adjusted for the average pup weight (approximately 20 g). Adjuvant control animals were treated with a 1:1 solution of Alhydrogel and saline, while saline controls were injected with an equal volume of 0.9% sterile saline. All treatments were brought up in sterile saline to reach a final total volume of 25 μL and were administered i.m. to the *vastus lateralis* muscle using a sterile 25-gauge needle. After treatment, pups were returned to their home cage where they remained with their dam for 2 or 5 days post-injection until they were euthanized for tissue collection.

### Blood and brain tissue collection

On P12 or P15, animals were deeply anesthetized with 4% isoflurane in oxygen. Blood samples were then collected via cardiac puncture followed immediately by transcardial perfusion with sterile phosphate-buffered saline (PBS). Blood was centrifuged (12,000×*g*, 4 °C, 10 min) to obtain serum, which was then stored at − 80 °C. Whole brains of animals randomly chosen for cytokine measurement analyses were quickly removed following transcardial PBS perfusion and, using a dissection scope and sterile surgical equipment, microdissected in PBS on ice to isolate the hippocampi, cortices, and cerebella. All tissues were snap-frozen in liquid nitrogen and stored at − 80 °C until further assayed. Animals randomly chosen for immunohistochemical analyses were anesthetized with 4% isoflurane in oxygen and subsequently perfused transcardially with 100-ml cold PBS at a rate of 15 ml/min using a Masterflex peristaltic pump (Cole Parmer, Vernon Hills, IL) followed by 100 ml of cold 4% paraformaldehyde (PFA) in PBS. Fixed tissues were removed, post-fixed in 4% PFA overnight, and then stored in 30% sucrose in PBS at 4 °C for 48 h. Fixed brains were snap-frozen in O.C.T. Compound (Sakura Finetek, Torrance, CA) and then sectioned into 10-μm-thick sagittal sections. Sections were stored at − 80 °C until further processed for immunohistochemical analyses.

### Cytokine and chemokine measurement

Prior to cytokine measurement, brain tissue samples were thawed and lysed in Bio-Plex cell lysis buffer containing protease and phosphatase inhibitors (Bio-Rad Laboratories, Hercules, California) and supplemented with 500 mM protease inhibitor phenyl-methylsulfonyl fluoride (PMSF; Sigma-Aldrich, St. Louis, Missouri). Briefly, tissues were homogenized in 200 μL (hippocampus) or 500 μL (cortex and cerebellum) of cell lysis solution using a polytron homogenizer for 10 s. The homogenate was then frozen for 10 min at − 80 °C, thawed, sonicated for 3 min, and then centrifuged at 4500 g for 4 min. Protein was quantified in the supernatant using Pierce BCA assay (Thermo Scientific; Rockford, Illinois), and samples were stored at − 80 °C until further analyzed.

Concentrations of 10 cytokines and chemokines were determined using a commercially available multiplex magnetic bead-based kit (Bio-Plex Pro™ Cytokine Reagent Rat Cytokine Assay; Bio-Rad Laboratories, Hercules, California) in accordance with the kit-specific protocols provided by Bio-Rad. The following cytokines and chemokines were measured: granulocyte macrophage colony-stimulating factor (GM-CSF), interferon gamma (IFN-γ), interleukin-1α (IL-1α), IL-1β, IL-4, IL-6, IL-10, IL-17, monocyte chemotactic protein-1 (MCP-1), and tumor necrosis factor α (TNF-α). Briefly, lyophilized rat cytokine standards were first reconstituted with either cell lysis buffer (brain samples) or the kit-provided standard diluent (serum samples), and a standard dilution series was made. Homogenized brain samples were run in duplicate at 1 mg/mL, whereas serum samples were run neat. Fifty microliters of samples, standards, and corresponding buffer blanks were incubated on a plate shaker at room temperature (RT) with antibody-coupled magnetic beads for 1 h. After a series of washes, biotinylated detection antibodies were added and incubated on a shaker at room temperature for 30 min. The reaction mixture was detected by the addition of streptavidin-phycoerythrin following a wash step and incubated on a plate shaker at room temperature for 10 min. Following a repeat of the washing step, beads were re-suspended in assay buffer for 30 s at room temperature on the plate shaker. Plates were read on a Bio-Plex 200 system (Bio-Rad Laboratories, Hercules, CA, USA) and analyzed using Bio-Plex Manager software (Bio-Rad Laboratories) with a five-parameter model used to calculate final concentrations and values (expressed in pg/mL). Reference samples were run on each plate to determine assay consistency. All wash steps were performed at room temperature using a Bio-Plex handheld magnetic washer (Bio-Rad Laboratories).

### Immunohistochemistry (IHC)

Sections were immunostained for glial fibrillary acidic protein (GFAP; 1:1000 dilution; Cell Signaling Technology, Danvers, Massachusetts), ionized binding adaptor protein 1 (IBA-1; 1:500 dilution; Wako Bioproducts, Richmond, Virginia), and CD68 (1:200 dilution; Serotec; Raleigh, NC). Antibody-antigen complexes were visualized using secondary antibodies labeled with Alexa Fluor 488, 568, or 647 (Molecular Probes; Eugene, OR), and slides were mounted in media containing 4′,6-diamidino-2-phenylindole (DAPI; Invitrogen, Carlsbad, CA) to identify cell nuclei. Images of the anterior cingulate (cortex) and dentate gyrus (hippocampus) were captured automatically by the ImageXpress Micro Widefield High Content Screening System (Molecular Devices, Sunnyvale, California) using thresholds set using region-matched saline controls. Images were acquired in an unbiased manner using the DAPI channel for each region. Average fluorescence intensity of the target antigen, as well as the number of GFAP immunopositive cells or the number of total IBA-1 immunopositive cells and percentage of IBA-1 immunopositive cells also immunopositive for CD68, was quantified from five fields per region of interest from three serial sections per brain for a total of 15 microscopic fields per brain. These values were averaged within a given animal for each brain region. A total of 3–5 animals were imaged per group.

### Experimental design and statistical analysis

Experimental groups were randomized. Different pups were used for cytokine analyses versus immunohistochemical analyses, and animals were randomly assigned to an outcome measure (cytokine measurement or immunohistochemistry) and day of collection (2 or 5 days post-challenge) prior to euthanasia. A total of 93 Lewis and 90 BN male and female offspring were included in this study (Luminex: 51 Lewis and 48 BN; IHC: 42 Lewis and 42 BN).

To assess cytokine levels, statistical analyses were performed using SPSS software (SPSS Version 22; IMB Corp., Armonk, NY); *p* values < 0.05 for two-tailed tests were considered statistically significant. Data graphs were created using GraphPad Prism (Version 6; GraphPad Software Inc., La Jolla, CA); all results are presented as mean ± SEM. All data were first assessed for the detection of outliers using the ROUT method, with Q set to 1%. As the distribution of the cytokine and chemokine concentration values were skewed, natural log transformations were used in order to approximate normality. For all values that were below the limit of detection (LOD), we assigned a value of LOD/2 prior to log transformation. A preliminary five-way ANOVA was conducted to determine the effects of sample type (cortex, cerebellum, hippocampus, or serum), cytokine/chemokine, treatment, offspring sex, and day of collection (P12 or P15). The initial five-way ANOVA results led us to run separate ANOVAs for each sample type and cytokine, as significant source × cytokine effects and interactions were noted for all variables. Therefore, individual three-way ANOVAs were conducted for each cytokine/chemokine and sample type, examining the effects of treatment, offspring sex, and day of collection on levels of cytokine/chemokines in each sample type. All post hoc pairwise comparisons of significant interactions within these three-factorial ANOVAs were Sidak-adjusted for multiple comparisons.

For IHC analyses, primary outcomes included average GFAP intensity, total GFAP count, number of IBA-1+ cells, and percentage of IBA-1+ cells co-labeled for CD68 in the hippocampus and cortex for each animal. Mixed-effects regression models, including animal-specific random effects, were used to assess the differences between three groups of animals (mixed immune, adjuvant, and saline) across the brain regions. Exploratory analysis indicated that a natural logarithmic transformation was needed for all outcomes other than colocalization to stabilize the variance and meet the underlying assumptions of the mixed-effects models. Due to zeroes for some outcomes, all values in those outcomes were shifted by 0.1 prior to taking the natural logarithm. Due to a high percentage of zeroes, colocalization was dichotomized to 0 or 1 (colocalization > 0) and a repeated measures logistic regression model was used. Day post-immunization (2 or 5), group (mixed immune, adjuvant, or saline), sex (male or female), and brain region (cortex, hippocampus) were all variables of interest in the models. Total cell count was included in all models as a covariate. Interactions between these variables were also considered. Akaike information criterion was used for model selection and Wald tests for comparing groups were used. Results for all outcomes other than colocalization are presented as geometric mean ratios between the immune challenge or adjuvant groups and the saline group. All IHC analyses were conducted using SAS version 9.4. Due to aspects of limited group numbers and the presence of numerous conditions, statistical comparisons between specific groups (i.e., strain) were not directly performed but were reported in parallel to relate findings.

## Results

### Sex- and region-specific differences in CNS cytokine expression at baseline

**Fig. 1:**
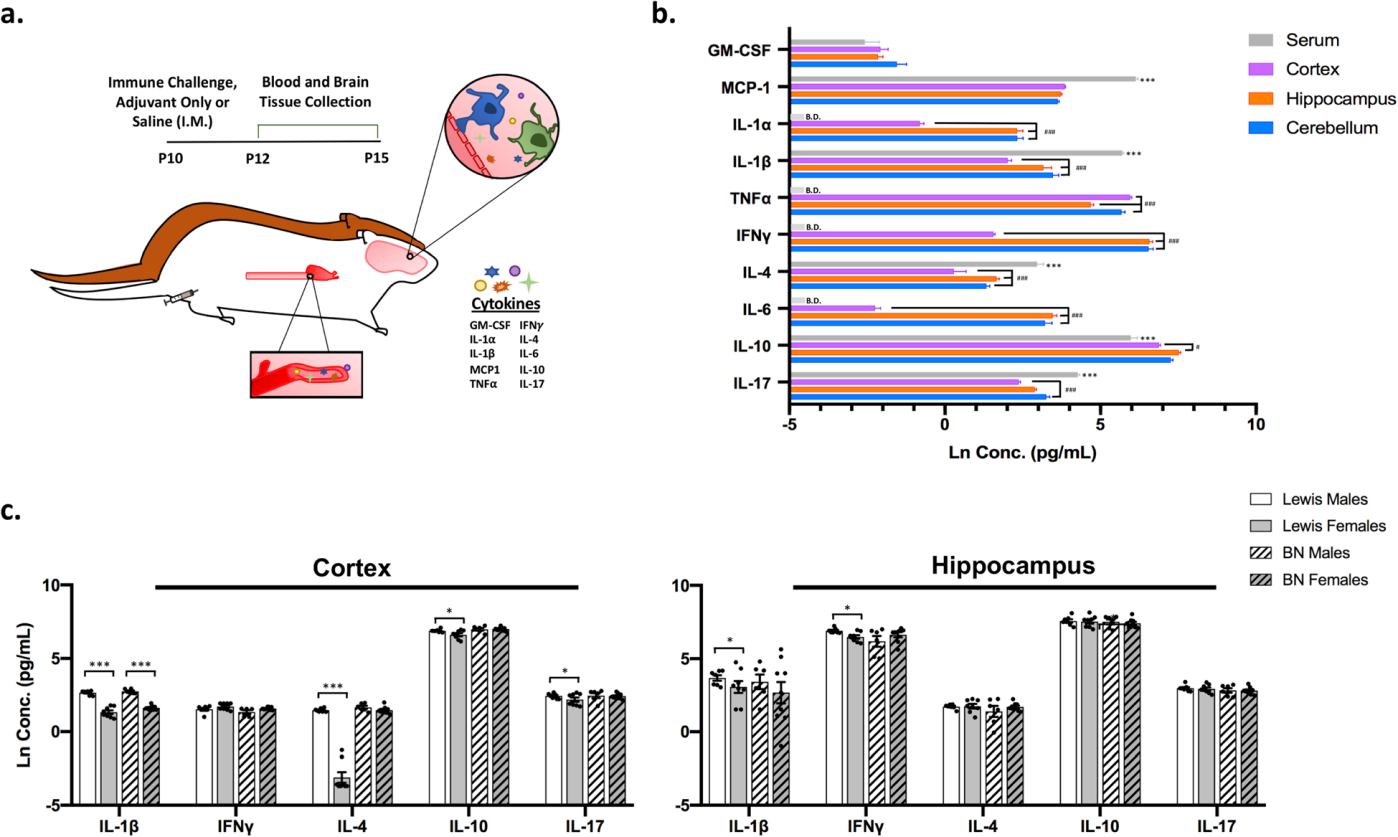
Sex- and region-specific differences in cytokine levels at baseline. Lewis and Brown Norway (BN) rats were injected i.m. with mixed immune challenge, adjuvant-only, or saline on P10. Samples were collected 2 or 5 days post-challenge and subjected to cytokine and chemokine profiling. **a** Illustration of experimental design with primary outcome measures. **b**, **c** Cytokine and chemokine levels under saline control conditions. **b** Cytokine levels compared within strains (BN (15; 6M, 9F), Lewis (15; 7M, 8F)) and between sexes; collapsed between time point of collection due to no differences observed. **c** Cytokine levels compared across region of collection; collapsed between sex, strain, and day of collection. *N* = 30 animals per region. Star (*) corresponds to comparisons between serum and all brain regions using the following scale: *p < 0.05, **p < 0.01, ***p < 0.001. Hashtag (#) represents comparisons solely between brain regions only using a similar scale. B.D. defined as below detection. All data are displayed as the natural log-transformed values with mean +/− SEM

Immune signaling is important for early development, and sex-specific differences have been evidenced in peripheral and CNS immune signaling under normal conditions [20]. Therefore, we wanted to examine whether cytokine levels exhibited sex-specific differences at baseline, under saline control conditions, during early postnatal development in Lewis and BN rat strains. To evaluate this, and all subsequent cytokine comparisons, we used a bead-based Luminex assay to assess the levels of a set of 10 analytes including a subset of Th-related cytokines, specifically IFN-γ, IL-4, IL-17, and IL-10, as well as inflammatory chemokines in peripheral blood and within different brain regions of experimental animals. Animals were exposed to peripheral immune challenge in adjuvant, adjuvant-only, or saline on P10, and samples were collected 2 and 5 days post-challenge in male and female Lewis and BN rats (Fig. 1a). Data presented for baseline sex comparisons were collapsed between both time points of collection, P12 and P15, as no statistically significant differences were observed between the two time points for saline control conditions.

Notable sex-specific differences in baseline cytokine levels were seen in the cortex and to a lesser extent the hippocampus. In the cortex, baseline sex differences were observed in several important Th-type cytokines, such as IL-4 (*p* < 0.001), IL-10 (*p* = 0.01), and IL-17 (*p* < 0.05) as well as IL-β (*p* < 0.001), with males exhibiting an increased level of these cytokines compared to females (Fig. 1b). Interestingly, these results were only true for Lewis rats and not observed in the BN rat strain, with the exception of higher IL-1β (*p* < 0.001) in male BN rats compared to females. When considering cytokine levels in the hippocampus, baseline sex-specific effects were more limited. Similar to the cortex, a significant difference was observed for IL-1β (*p* = 0.01) in Lewis rats. Additionally, a sex-specific increase in IFN-γ (*p* < 0.05) was also seen in the hippocampus of Lewis rats, with males displaying a higher level of IFN-γ than females (Fig. 1b).

Minimal sex-specific differences were seen in serum cytokine levels at baseline, with the only significant finding being an effect of sex on the level of MCP-1 (monocyte chemoattractant protein 1) in Lewis rats, with males displaying a greater level of MCP-1 than females (*p* = 0.004; Additional file 1: Figure S1A). No sex-specific differences were observed in the cerebellum under saline control conditions (Additional file 1: Figure S1D). In multiple brain regions, baseline differences in GM-CSF levels were noted but data were not included in the final analysis due to several samples having values below the LOD, thus skewing group differences (Additional file 1).

Brain region-specific differences in the level of several cytokines were also noted. In serum, levels of the majority of analytes were found to be significantly different than concentrations of these same cytokines measured in brain regions of the corresponding animals (Fig. 1c). Specifically, the levels of MCP-1 (*p* < 0.001), IL-1β (*p* < 0.001), IL-4 (*p* < 0.001), and IL-17 (*p* < 0.001) were observed to be higher in serum than the cortex, hippocampus, and cerebellum, while the level of IL-10 (*p* < 0.001) appeared lower in the serum compared to selected brain regions (Fig. 1c).

Differences were also observed when comparing cytokine levels between brain regions with main findings including lower levels of many analytes in cortical lysates. The levels of IL-1α (*p* < 0.001), IL-1β (*p* < 0.001), IFN-γ (*p* < 0.001), IL-4 (*p* < 0.001), and IL-6 (*p* < 0.001) were seen to be lower in the cortex, compared to both the hippocampus and cerebellum (Fig. 1c), while IL-10 (*p* < 0.05) was significantly lower in the cortex compared to the hippocampus only, and cortical IL-17 (*p* < 0.001) was less than that measured in the cerebellum. Additionally, the hippocampal level of TNF-α was significantly lower in the hippocampus compared to other brain regions (*p* < 0.001; Fig. 1c).

### Effect of peripheral immune challenge on serum cytokines and chemokines

**Fig. 2:**
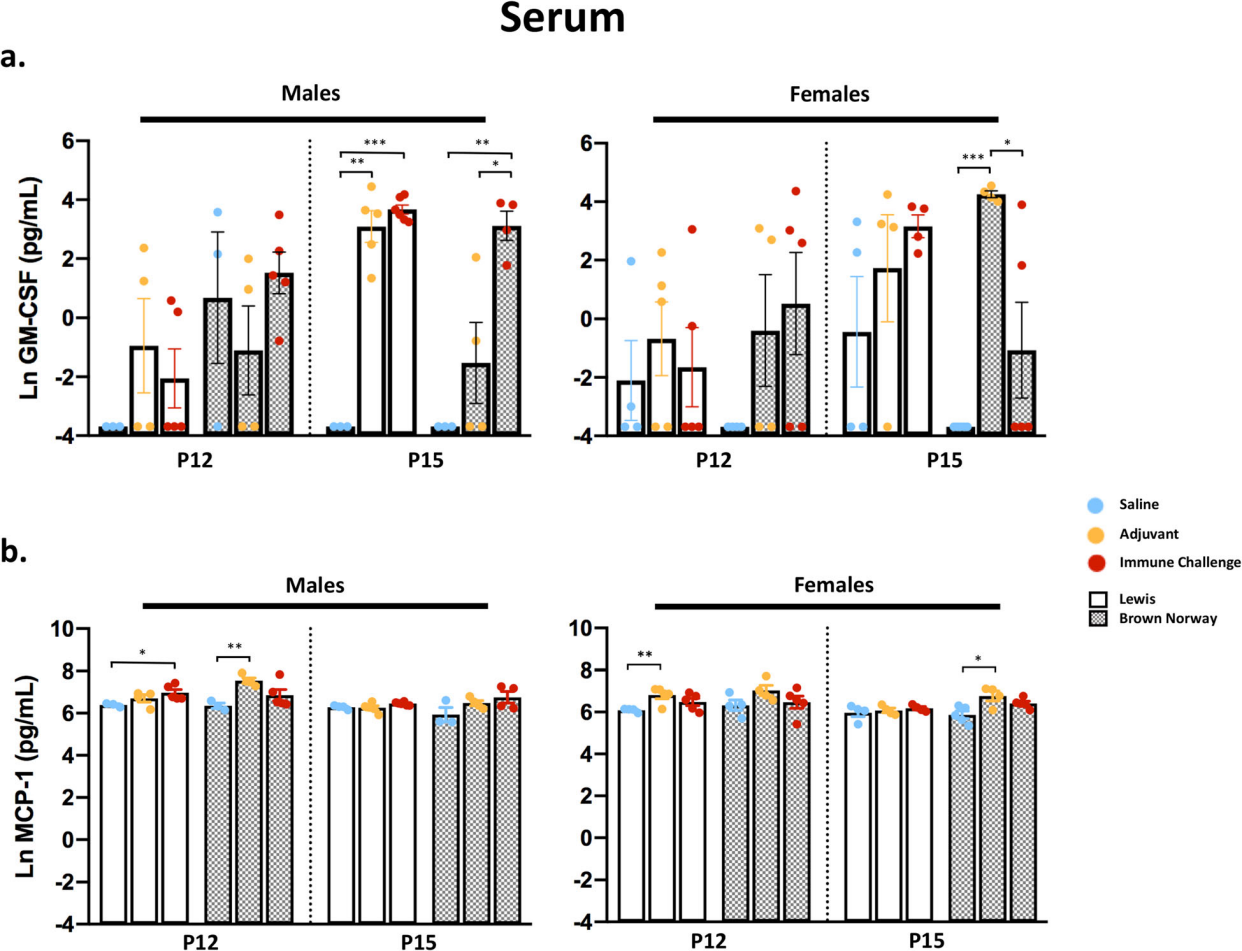
Elevated serum cytokines in response to mixed immune challenge or adjuvant. Serum cytokine and chemokine levels of GM-CSF (**a**) and MCP-1 (**b**) following immune stimulus represented by day of collection (P12/P15), strain (Lewis/BN), and sex. Data presented as natural log-transformed values with mean +/− SEM. *N* = 3–6 animals per condition; *p < 0.05, **p < 0.01, ***p < 0.001

Next, to broadly characterize the innate and adaptive immune responses to an early postnatal peripheral immune challenge, we assessed post-challenge cytokine levels between strain, sex, region, and time point of collection as described above and outlined in Fig. 1a. Consistent with the age of the rat pups, the effect of treatment on the serum cytokine response was relatively mild (Additional file 2: Figure S2A and Additional file 3: Figure S3A). Over half of the serum samples had levels of IFN-γ, IL-1α, IL-6, and TNF-α below the LOD, and these cytokines were therefore excluded from further analysis. For serum analytes that were above the LOD and were found to be differentially regulated in response to treatment, effects were broadly similar across strains with some sex-specific skewing. For GM-CSF, significant main effects of treatment were seen in both Lewis and BN rat strains (Additional file 2: Figure S2A). Specifically, post hoc testing revealed sex-dependent increases in the level of GM-CSF in serum 5 days post-challenge in males of both strains (Lewis, *p* < 0.001; BN, *p* = 0.005; Fig. 2a), with significant increases also seen under adjuvant conditions in male Lewis rats (*p* = 0.001). In contrast, female BN rats exhibited significant increases in GM-CSF in response to adjuvant treatment compared to saline controls at the same time point (*p* < 0.001; Fig. 2a). Of note, a significantly higher level of GM-CSF was seen under adjuvant conditions compared to immune challenge in female BN rats at 5 days post-challenge (Fig. 2a). However, this was the only instance of such a finding in the study and is likely due to several serum GM-CSF values falling below the level of detection in these animals. A main effect of treatment was observed in the level of the chemokine MCP-1 in both Lewis and BN rats (Additional file 2: Figure S2A). Higher levels of MCP-1 were detected under adjuvant-only conditions at 5 days post-challenge in female BN rats (*p* = 0.02) and at 2 days post-challenge in female Lewis rats (*p* = 0.001), or 2 days following mixed immune challenge in Lewis males (*p* = 0.016; Fig. 2b).

### Innate immune cell-related cytokine response in the CNS

**Fig. 3:**
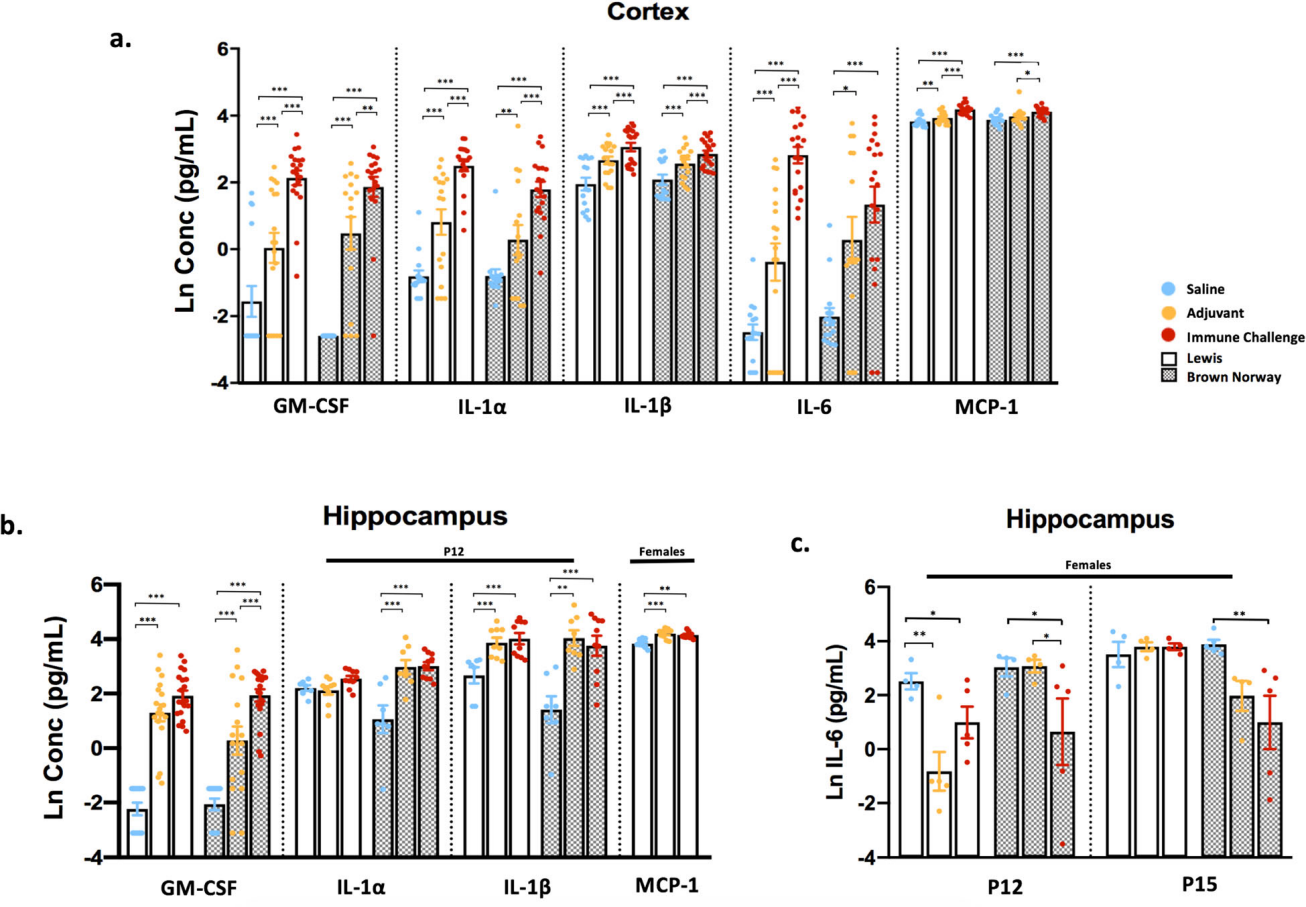
Peripheral immune stimulation broadly upregulates innate cytokine levels in the cortex and hippocampus. Cortical, hippocampal, and cerebellar lysates were collected from rats 2 or 5 days following exposure and subjected to cytokine and chemokine analysis. **a** Concentrations of innate cytokines in the cortex, compared between experimental conditions. Data are collapsed between sex and day due to minimal differences seen; *N* = 15–20 per condition. **b**, **c** Cytokine levels from hippocampal lysates; black solid bars above certain analytes specify time point or sex-specific conditions. **b** Levels of several innate cytokines: GM-CSF collapsed between sex and day (*N* = 13–20 per condition), IL-1a, IL-1B shown at P12 only and MCP-1 in females collapsed between day (*N* = 7–10 per condition). **c** Hippocampal IL-6 levels across treatment and strain in female rats; *N* = 4–5 per condition. Data represent mean +/− SEM, *p < 0.005, **p < 0.01, ***p < 0.001

To understand the CNS immune response to an acute peripheral immune challenge, we evaluated cytokine and chemokine levels in tissue lysates from the cortex, hippocampus, and cerebellum of male and female Lewis and BN rats. The most striking results and greatest inflammatory response to the mixed immune challenge were seen in the cortex. In both male and female Lewis and BN rats, a significant main effect of treatment was observed for several cytokines associated with the innate immune response in the cortex at 2 and 5 days following treatment (Additional file 2: Figure S2B). Specifically, IL-1α, IL-1β, and IL-6 were significantly upregulated following either peripheral immune challenge or adjuvant-only exposure in both strains (Fig. 3a). Similar effects of treatment were noted for GM-CSF and MCP-1, both important innate immune cell recruitment and activation molecules (Fig. 3a). Representative cortical innate immune cytokine data are collapsed across sex and time point of collection as a response to treatment appeared similar between these conditions. The only exception to this pattern was the lack of increased MCP-1 expression in the cortex in male BN rats exposed to either peripheral immune challenge or adjuvant-only (Additional file 3: Figure S3B).

In the hippocampus, a main effect of treatment was also seen for the majority of cytokines (Additional file 2: Figure S2C). A significant increase in GM-CSF compared to saline controls was observed at both 2 and 5 days post-challenge across experimental conditions, strains, and sexes (Fig. 3b, Additional file 3: Figure S3C). In both sexes, significant increases in IL-1α (*p* < 0.05) and IL-1β (*p* < 0.001) were noted in BN rats, while a significant increase in IL-1β (*p* < 0.001), but not IL-1α, was observed in Lewis rats at 2 but not 5 days post-challenge (Fig. 3b). Elevated levels of MCP-1 were seen only in female Lewis rats at 2 days (*p* = 0.01) and 5 days (*p =* 0.034) post-challenge (Fig. 3b). Interestingly, immune challenge decreased hippocampal IL-6 relative to saline control in female rats of both strains (*p* < 0.05; Fig. 3c). Other cytokines, such as IL-1α and IL-1β, were also decreased by peripheral immune challenge or exposure to adjuvant-only at 5 days post-challenge (Additional file 3: Figure S3C). The effect of peripheral immune challenge on the cytokine response in the cerebellum was both weak and varied (Additional file 2: Figure S2D), with the exception of a significant increase in GM-CSF, similar to that seen in the hippocampus and cortex (Additional file 3: Figure S3D).

### Th-type cytokine responses in the CNS

**Fig. 4:**
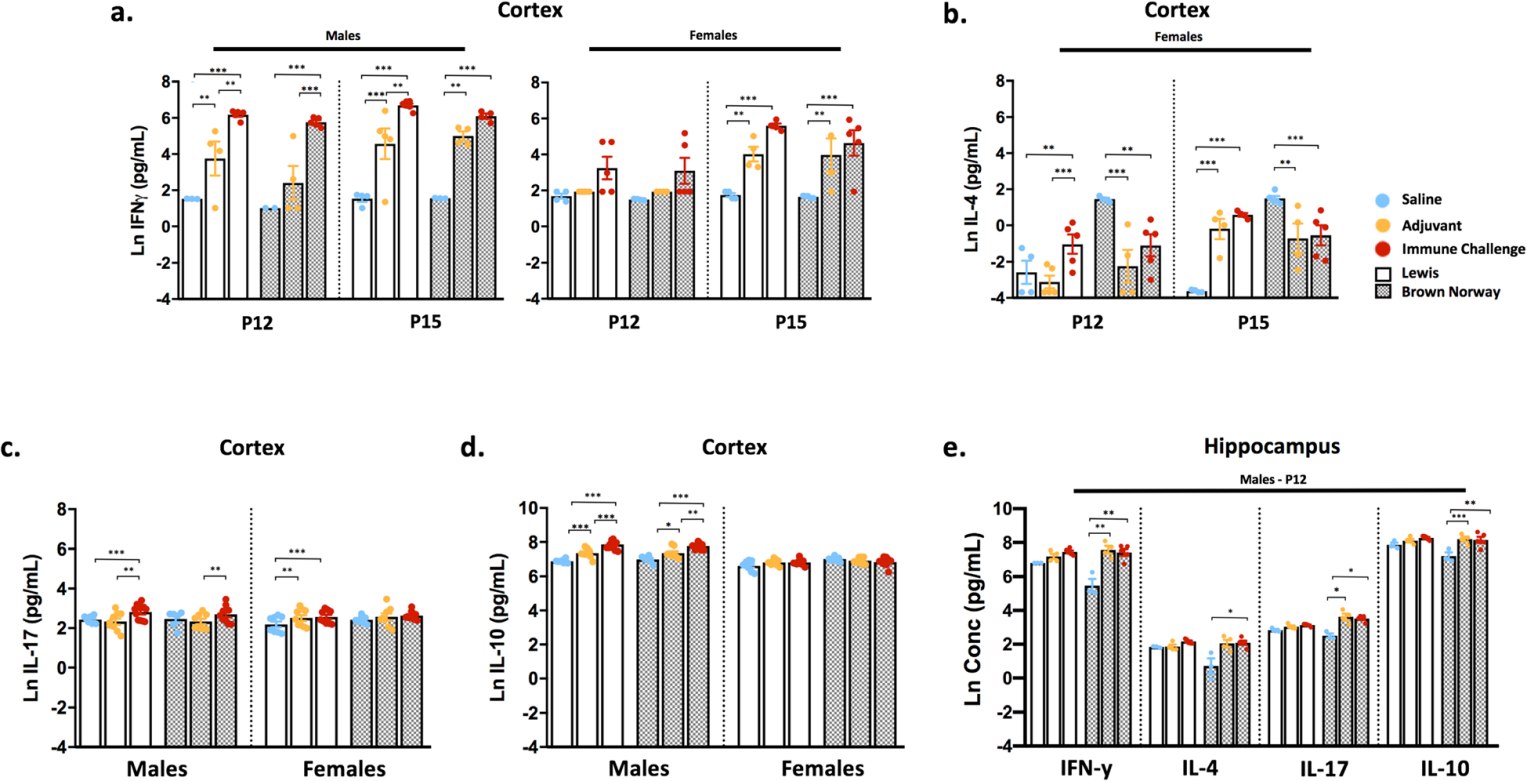
CNS Th-type responses to peripheral immune stimulation are sex- and strain-specific. Th-type cytokine responses were evaluated in brain lysates. Cortical IFN-γ; *N* = 3–6 per condition (**a**), IL-4; *N* = 3–5 per condition (**b**), IL-17; *N* = 7–11 per condition (**c**) and IL-10; *N* = 7–10 per condition (**d**) levels. IL-4 displayed only in female rats due to no differences seen in males; IL-10 and IL-17 levels collapsed between day. **e** Hippocampal Th-type cytokine levels in male rats at 2 days post-challenge; *N* = 3–5 per condition. Data represent mean +/− SEM, *p < 0.005, **p < 0.01, ***p < 0.001

Due to evidence suggesting that the immune responses in Lewis and BN rats are skewed toward a Th1- or Th2-specific response, respectively [21, 22], a primary aim of this study was to evaluate the contribution of a different genetic immune background on the response to immune challenge. In cortical lysates from both strains, a main effect of treatment was observed for all canonical Th-type cytokines measured (Additional file 2: Figure S2B). Interestingly, the level of IFN-γ (a major Th1 cytokine) in the cortex following mixed immune challenge was significantly elevated in males of both strains (*p* < 0.001), with similar effects at 2 and 5 days post-challenge (Fig. 4a). In female rats of either strain, cortical IFN-γ levels were significantly increased at 5 days (*p* < 0.01) but not at 2 days post-challenge (Fig. 4a). In contrast to the cortical response, peripheral immune challenge had little or no significant effect on IFN-γ levels in the hippocampus and cerebellum of Lewis or BN rats (Additional file 3: Figures S3C and D). Striking strain and sex differences were seen in the response to immune challenge in cortical levels of IL-4, an indicator of Th2-type responses. Specifically, peripheral immune challenge significantly increased cortical IL-4 levels in female Lewis rats at 2 days (*p =* 0.003) and 5 days (*p <* 0.001) post-challenge, while exposure to adjuvant-only treatment only resulted in elevated cortical IL-4 levels at 5 days post-exposure (*p <* 0.001; Fig. 4b). In contrast, compared to saline controls, cortical IL-4 levels were significantly decreased in female BN rats in response to mixed immune challenge (*p <* 0.01) or adjuvant (*p <* 0.01), or unchanged in male rats of either strain (Fig. 4b; Additional file 3: Figure S3B). Coincident with these responses, post hoc analysis revealed a lack of effect of either immune challenge or adjuvant-only on IL-17 levels in the BN rat cortex at both 2 and 5 days post-challenge. However, cortical IL-17 levels were significantly elevated compared to controls in response to mixed immune challenge in male Lewis rats (*p <* 0.001) and in response to either mixed immune (*p <* 0.05) or adjuvant treatment (*p <* 0.05) in female Lewis rats (Fig. 4c). Cortical IL-10 levels were similar between strains, although significantly increased levels of IL-10 in response to experimental manipulation were seen only in male rats, with no apparent effects in females (Fig. 4d). Data for cortical IL-17 and IL-10 levels were collapsed between day of collection due to minimal differences seen for those two analytes over time (Additional file 3: Figure S3B).

In the hippocampus, male BN rats exhibited significant increases in IFN-γ (*p <* 0.001), IL-4 (*p =* 0.035), IL-10 (*p =* 0.037), and IL-17 (*p <* 0.001) in response to treatment at 2 days post-immune challenge (Fig. 4e). Interestingly, there were no significant increases in the levels of these cytokines in the hippocampus of female BN rats or either sex of Lewis rats (Additional file 3: Figure S3C). While a main effect of treatment on Th-type responses in the cerebellum was apparent under certain conditions (Additional file 2: Figure S2D), post hoc analysis revealed effects of treatment on cytokine production in the cerebellum to be largely non-significant across most conditions (Additional file 3: Figure S3D).

### Evaluation of CNS cellular immune response

To understand the effects of mixed immune challenge or adjuvant-only exposure on the brain-specific cellular response, the number of GFAP immunopositive cells and the average intensity of GFAP immunofluorescence were evaluated as indicators of astrogliosis, whereas the total number of IBA-1 immunopositive cells and percentage of IBA-1 immunopositive cells also immunoreactive for CD68 were quantified to assess the microglial response.

**Fig. 5:**
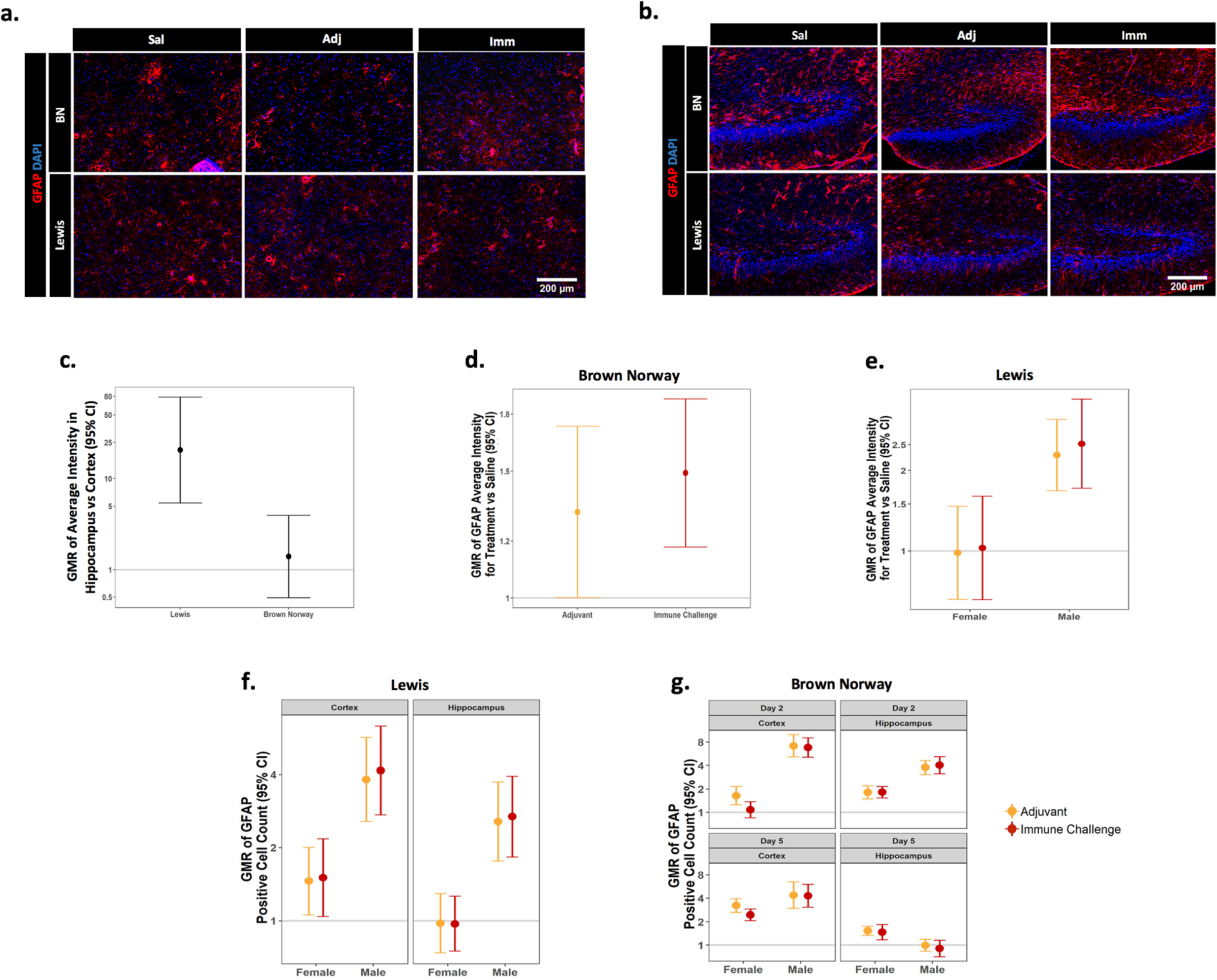
Sex- and strain-specific increases in the average intensity of GFAP immunoreactivity and numbers of GFAP immunopositive cells. GFAP immunoreactivity was assessed at 2 and 5 days post-exposure. Representative photomicrographs of GFAP immunoreactivity and DAPI labeling in the cortex (**a**) of female rats and the hippocampus (**b**) of male BN and female Lewis rats; Sal=saline, Adj=adjuvant-only, Imm=immune challenge. **c** Geometric mean ratio (GMR) of GFAP average intensity in response to immune challenge, adjuvant-only, or saline control conditions in the hippocampus versus the cortex within each strain; collapsed between sex and day of collection (*N* = 42 per strain; BN(19Imm, 19Adj, 4Sal); Lewis(16Imm(8M/8F), 17Adj(9M/8M), and 9Sal(5M/4F)). GMR plots of GFAP average intensity in BN (**d**) and Lewis (**e**) rats collapsed between day and region, as well as GFAP-positive cell counts in Lewis (**f**) and BN (**g**) strains of each treatment relative to saline control collapsed between the day of collection in (**f**). Error bars represent 95% CI; a CI not including the normalization line (line at 1) indicates a significant difference between brain regions (**c**) or the treatment and saline controls (**d**–**g**) at *p* ≤ 0.05

GFAP is an intermediate filament expressed mainly in astrocytes that is upregulated under conditions of hypertrophy and activation [24]. In both the cortex and hippocampus, GFAP average fluorescence intensity was significantly increased within both strains at 2 and 5 days post-challenge. In Lewis rats, across all conditions, the average intensity of GFAP immunoreactivity was significantly higher in the hippocampus than in the cortex, whereas no significant regional differences were observed in BN rats (Fig. 5a–c). In addition to brain region-specific strain differences, sex-specific effects of both mixed immune challenge and adjuvant-only exposure on average intensity of GFAP immunofluorescence were observed. While exposure to either treatment significantly increased the average intensity of GFAP immunofluorescence in male and female BN rats across brain regions at 2 and 5 days post-challenge (Fig. 5d), exposure to either treatment in Lewis rats significantly increased the average intensity of GFAP immunofluorescence in males only (Fig. 5e). Due to best-fit statistical modeling used for IHC analysis, conditions were collapsed between groups when no differences were observed. With regard to GFAP intensity as noted here, a similar response to immune challenge was seen across the brain region within a rat strain.

A significant increase in the number of GFAP immunopositive cells was also seen in response to mixed immune challenge or adjuvant-only exposure, but these effects varied between brain regions and strains. In the cortex, GFAP immunopositive cell counts were increased following mixed immune challenge or adjuvant-only exposure in both strains at 2 and 5 days post-challenge (Fig. 5f, g) with the exception of female BN rats that exhibited no significant change in GFAP immunopositive cell counts at 2 days post-mixed immune challenge (Fig. 5g). In contrast, more consistent sex and strain differences were seen in the hippocampus. In Lewis rats, males exhibited a significant increase in the number of GFAP immunopositive cells in the hippocampus 2 and 5 days post-challenge following mixed immune challenge or adjuvant-only exposure, whereas female Lewis rats showed no differences in response to either challenge (Fig. 5f). In the BN strain, the numbers of GFAP immunopositive cells in the hippocampus significantly increased in both sexes 2 and 5 days post-challenge, with the exception of male BN rats that exhibited no effect at 5 days post-challenge (Fig. 5g).

**Fig. 6:**
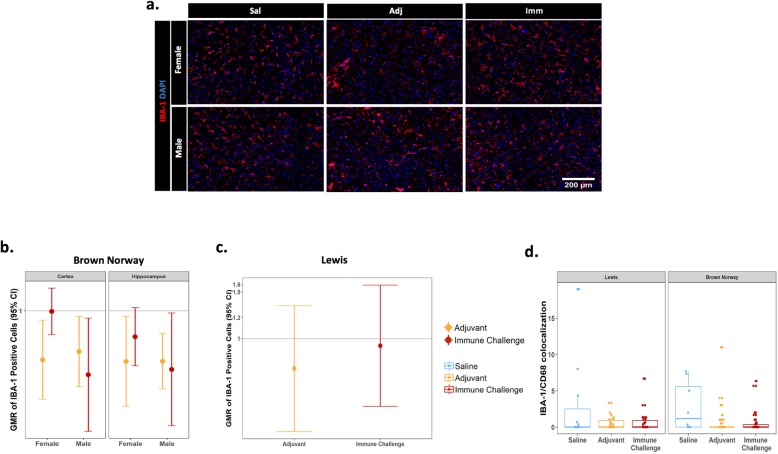
Minimal effects of treatment on IBA-1 immunoreactivity. The number of IBA-1 immunopositive cells was quantified in the cortex and hippocampus in response to treatment. **a** Representative photomicrographs of IBA-1 immunoreactivity and DAPI labeling in the cortex of BN rats; Sal=saline, Adj=adjuvant-only, Imm=immune challenge. Geometric mean ratio (GMR) plots displaying the number of IBA-1 immunopositive cells for treatment versus saline control in BN (*N* = 40; 18Imm(10M/8F), 18Adj(9M/9F), 4Sal(2M/2F) (**b**), and Lewis (*N* = 42; 15Imm, 15Adj, 7Sal) (**c**) rat strains. Data for IBA-1 immunopositive cell counts expressed as ratio over saline control conditions. Error bars represent 95% CI; a CI not including the normalization line (line at 1) indicates a significant difference between the treatment and saline controls at *p* ≤ 0.05. **d** Quantification of IBA-1/CD68 colocalization within strains and across treatment conditions, data expressed as boxplots illustrating the distribution of data points for each animal and the interquartile range

The cellular neuroinflammatory response was further evaluated by immunostaining for IBA-1 in the cortex and hippocampus. IBA-1 is a pan-macrophage/monocyte marker used broadly in the brain to identify microglia [25]. Interestingly, in contrast to increased brain GFAP immunofluorescence intensity and number of GFAP immunopositive cells seen across many conditions, the number of IBA-1 immunopositive cells in the cortex and hippocampus were either unchanged or significantly decreased in these regions in response to mixed immune challenge or adjuvant-only exposure, compared to saline controls (Fig. 6a, b). In BN rats, a reduction in the number of IBA-1 immunopositive cells was observed in the cortex and hippocampus of male rats exposed to either mixed immune challenge or adjuvant-only and in the cortex and hippocampus of females treated with adjuvant-only (Fig. 6b). In contrast, there was no difference in the number of IBA-1 immunopositive cells in female BN rats exposed to mixed immune challenge or Lewis rats under either treatment condition compared to saline controls (Fig. 6b, c).

Additionally, colocalization of CD68 immunoreactivity with IBA-1 was used as a measure of microglial activation in the brain, as CD68 is a lysosomal marker used broadly to indicate phagocytic activity in macrophages [26]. Following the analysis of the response to either mixed immune challenge or adjuvant exposure, there were no significant differences between treatment conditions, strain, or sex with respect to IBA-1/CD68 colocalization (Fig. 6d).

## Discussion

The physiologic importance of the interplay between the immune and nervous systems in neurodevelopment has gained recognition in recent years, and immune molecules are increasingly implicated as important in neurogenesis, cortical development, and neurodevelopmental disorders [27–30]. Despite this, knowledge regarding the CNS response to a peripheral immune challenge during early postnatal development is limited in scope. Numerous studies have investigated perinatal immune signaling and CNS development using iterations of the maternal immune activation (MIA) paradigm (reviewed in [31]). This is likely due to the growing body of literature on the topic, clinical relevance, and thorough characterization of the methodology [32]. Although, recent research has suggested, perhaps unsurprisingly, that MIA-induced effects on brain circuit-specific function are dissociable from those effects induced by early postnatal immune challenge, with contrasting results on glutamatergic and GABAergic signaling respectively [33]. Therefore, it is of value to develop a deeper knowledge regarding how acute peripheral immune stimulation, postnatally, may influence the production of immune effector molecules and activation of glial cells in the CNS. The latter point is particularly important as altered neural function due to perinatal immune activation may depend partly on early glial priming, contributing to long-term functional alterations in these cells [5, 6, 30].

An important aspect of our study design is the developmental window during which the acute mixed immune challenge occurred. Prior studies comparing the timeline of rat and human development suggest that preweaning ages in rats correspond to roughly the first year of life in humans [16, 17]. Therefore, to mirror the immune challenges a human infant may face in early life, we chose to expose rats on P10—a time of early postnatal development when hematopoiesis is shifted from the fetal liver and spleen to the bone marrow, and the lymphoid architecture begins to take shape [34]. Furthermore, in rodent development, P10 is a time shortly after the early critical period for sexual differentiation of the brain [35] and represents an age of peak brain growth in rats [18]. These factors are important for proper interpretation of sex- and age-specific comparisons in this study.

To characterize peripheral and central responses, we collected serum and brain samples from Lewis and BN rats at 2 and 5 days post-exposure to immune challenge, adjuvant-only, or saline. These time points were chosen to correspond to an early stage, 2 days post-exposure, where innate immune mechanisms would be dominant, and at a later stage, 5 days post-exposure, where adaptive immune responses would potentially be active [36]. Importantly, development of the adaptive immune system in rodents is similar to humans in that perinatal lymphocyte numbers and proliferative responses in lymphoid organs are low [37]. Further evidence suggests that germinal center formation is absent at P10 in rats and, subsequently, they are thought to be incapable of mounting a proper primary immune response [38]. Therefore, it is not surprising that many cytokine levels were below the level of detection in the peripheral blood. However, it is notable that the cytokines IL-4, IL-10, and IL-17 were detectable in serum, albeit with no significant differences between experimental groups or rat strain. Relevant here, and known to be similar between species, evidence shows that early postnatal Th-type responses in rats and other mammals are skewed toward Th2 [39, 40]. Furthermore, studies in neonatal mice using exposure to various inoculation components resulted in a decreased IgG (immunoglobulin G) 2a/IgG1 ratio compared to similarly exposed adult animals [41]. These data support a bias of a Th2-type versus Th1-type response during the neonatal period. The inability to detect serum IFN-γ in our samples while observing measurable IL-4 and IL-10 levels may lend further credence to this potential skewing.

In contrast to the modest peripheral response, the cytokine and chemokine responses within the CNS were significantly more robust, with the most dramatic effects noted in the cortex. While the cytokines IL-1α and IL-6 were not detectable in the serum, they were markedly elevated in the brains of rats of both strains following the peripheral immune stimulation or adjuvant-only exposure, compared to saline control animals. Of importance, IL-1 and IL-6 are known to modulate glial responsiveness and are suggested to be crucial for glial proliferation and the release of important trophic factors to support brain plasticity [42, 43]. These current data may support this finding, as male BN rats, which exhibited no significant differences in IL-1 or IL-6 levels in the hippocampus 5 days post-treatment, also exhibited no difference in the number of GFAP immunopositive cells in the same region at the same time point in these animals. Whereas, at other time points and between sexes, there were significant differences in relevant cytokine levels and GFAP immunopositive cell numbers. It is important to note that the relationships described here and elsewhere regarding glial activation and cytokine levels are partly speculative as direct comparisons between GFAP or IBA-1 reactivity and specific cytokines were not conducted due to the large number of groups already being compared. Another innate cytokine significantly upregulated in the brain within the majority of treatment groups was GM-CSF, long known as a regulator of macrophage differentiation and more recently believed to play a role in myeloid cell to lymphocyte communication [44, 45]. This may suggest expansion or activation of the resident macrophage population. However, no significant differences in the number of IBA-1 immunoreactive cells were noted between experimental conditions and among different brain regions in Lewis rats, whereas significant decreases in IBA-1 immunopositive cell numbers were observed in the cortex and hippocampus of BN rats, with the exception of female BN rats exposed to mixed immune challenge. MCP-1, a chemokine important for glial differentiation and motility [46], was also significantly elevated in response to treatment. However, the MCP-1 response was sex and strain-specific, with no observable relationship to changes in GFAP or IBA-1 immunostaining. Our findings that GM-CSF and MCP-1 levels did not appear to relate to the immunohistochemical results of CNS cellular immune activation are surprising due to their putative role in potentiating glial responses [47]. Possible explanations for this discrepancy could be the developmental stage of the animals at the time of exposure and sample collection, or that these molecules may act as signals to recruit other immune cells to the CNS compartment. As limitations in this study, we did not investigate the possibility of peripheral immune cell infiltration in the brain, blood-brain barrier permeability, or the cellular source of the cytokines/chemokines. These aspects are certainly important to determine mechanistic aspects of the immune dysregulation seen but were outside the scope of the initial study aims and are definite points for future investigation.

In support of the concept that cytokine and chemokine signaling is important for glial regulation and neurodevelopment under normal conditions, levels of most cytokines and chemokines within the CNS of both Lewis and BN rats were already strikingly high under saline control conditions. This result appeared across the brain regions surveyed, with some variation at the level of single analytes. For example, IL-4, IL-10, and IL-17 were detected at surprisingly high levels throughout the brain at baseline, whereas levels of IL-6 were robust in the hippocampus and cerebellum, yet barely detectable in the cortex. These findings corroborate previously published data also observing appreciable levels of cytokines across brain regions at baseline during early postnatal development and specifically validate outcomes seen in our dataset such as lower IFN-γ in the cortex compared to the hippocampus in the second postnatal week [48]. While of interest, the biologic significance behind this brain region-specific difference in IFN-γ and other cytokines does not seem to be evident in the existing literature and will be important for further study. Additionally, our study suggests that cytokine levels at baseline appear to vary widely as a function of sex or strain. These data strongly support previous findings that immune molecule signaling in the CNS is active during normal neurodevelopment [49] and suggest consideration of sex and strain differences in the design of neuroimmunological experiments.

While innate-like cytokines and chemokines within the CNS showed broadly similar patterns across strain and sex, CNS Th-type cytokine responses varied greatly between Lewis and BN rats. These two rat strains were chosen for comparison due to previous knowledge of the susceptibility or resistance of either strain to the development of Th-subset specific disorders [50, 51]. However, the basis of this skewing may not only involve the CD4+ T cell compartment, but also CD8+ T cells and mast cells [21, 52, 53]. Given these findings, it may be anticipated that this immune response skewing could extend to the CNS as well. Using IFN-γ as a crude marker of a Th1-type response, our results suggest that these two strains do not differ greatly in Th1-type responses within the CNS. However, when evaluating IL-4 levels as a readout of a Th2-type response, the results were dramatically different. In the cortex, where the most robust cytokine responses were noted, female Lewis rats exhibited elevated levels of IL-4 in response to mixed immune challenge or adjuvant, whereas a significant decrease in IL-4 was noted in female BN rats under the same treatment conditions. No significant treatment-related differences in the level of IL-4 were observed in male rats of either strain. These results seem in opposition of what might be expected with Lewis rats exhibiting a greater IL-4 response and BN rats showing no changes or decreases in IL-4 levels in cortical lysates. However, when considering that we also observed sex and strain differences in other Th-subtype cytokines, this outcome suggests several non-mutually exclusive possibilities. First, as supported by the current study, the CNS cytokine response is likely different than the concomitant peripheral immune response. Second, it could be that genetic immune skewing between Lewis and BN strains cannot be classified into a defined Th-subset category. Finally, sex may be a greater determinant than genetic background when considering CNS immune skewing. Inherent differences in Th1/Th2 skewing between sexes have been previously proposed, with females skewed toward a Th2 dominant response [54]. Our data are consistent with this last point, at least with regard to IL-4 levels in the CNS.

These cytokine results, coupled with IHC evidence of significant increases in GFAP reactivity in female BN rats, may suggest a model in the cortex in which decreased IL-4 levels are related to astrocytic activation. In support of this concept, recent work has shown that pretreatment with IL-4 prior to peripheral LPS exposure abolishes LPS-induced astrocytic activation in the cortex of mice, as measured by GFAP immunoreactivity and iNOS expression [55]. Additionally, under many conditions, IL-4 responses are characterized as anti-inflammatory in nature and may act as a growth or repair responses in the brain [56, 57]. Therefore, it is plausible that IL-4 levels in the brain play an important role in regulating cellular inflammatory status, at least in regard to astrocytic activation.

Surprisingly, peripheral immune challenge or adjuvant exposure alone either had no effect or significantly decreased the number of IBA-1 immunopositive cells in the cortex and hippocampus. Additionally, no significant effects were noted upon assessment of IBA-1 colocalization with CD68. This is interesting as we saw a robust increase in GFAP immunopositive cells and expression levels across most conditions. The reasoning for this could lie once again in the developmental time period of exposure, as microglia undergo distinctive rounds of maturation during the perinatal period [58, 59]. In support of this reasoning, recent work has demonstrated that peripheral LPS challenge in P14 mice resulted in a significant increase in GFAP but not IBA-1 reactivity in the hippocampus [60]. More importantly, opposite effects were observed when adult animals were subjected to the same treatment, with pronounced increases in IBA-1 reactivity but not GFAP reactivity [60]. Furthermore, a separate group found that glial activation occurred sequentially in response to a systemic immune challenge, with microglial activation occurring shortly after exposure and induction of astrocytic activation occurring in a delayed manner [61]. These studies suggest that glial activation is tightly regulated both temporally and spatially.

## Conclusion

The current study supports existing evidence that immune signaling molecules are highly upregulated in the brain following a peripheral immune challenge. Additionally, it emphasizes the influence of factors such as sex and genetic background on the cytokine and chemokine response, as well as astrogliosis and microgliosis within the brain. Interestingly, we observed high concentrations of various cytokines in the CNS under baseline conditions, the levels of which also varied significantly depending on strain, sex, and brain region. While this study provides a thorough characterization of the CNS immune response to a peripheral immune challenge, taking into account a broad number of factors, further study is needed to provide mechanistic support for how this cytokine/chemokine signaling and cellular activation may shape brain development.

## Additional files

Additional file 1: Baseline cytokine results. Analysis of cytokine levels in response to saline-only conditions. Results represent cytokine concentrations in the serum (**A**), cortex (**B**), hippocampus (**C**), and cerebellum (**D**) of male and female Lewis (*N*=15; 7M, 8F) and BN (N=15; 6M, 9F) rats. Data represent mean +/- SEM, collapsed between day of collection. Value b.d. represents analytes where >50% of samples were below the level of detection and excluded from analysis; *p<0.005, **p<0.01, ***p<0.001. (JPG 1626 kb)

Additional file 2: Main effect of treatment on cytokine levels. Results of ANOVA analyses considering a main effect of treatment on cytokine and chemokine levels in the serum (**A**), cortex (**B**), hippocampus (**C**), and cerebellum (**D**) of Lewis and BN rats. Red coloring denotes a significant finding (*p*<0.05), while pink coloring represents a trending result (0.05<*p*<1). (JPG 1038 kb)

Additional file 3: Total cytokine and chemokine protein analyses of serum and brain lysates. Multi-factorial ANOVA analyses were conducted; *p*-values displayed here reflect Sidak-adjusted values for multiple comparisons. Tabular results of comparative cytokine and chemokine analyses between treatment conditions and within day of collection, sex, and strain; split between results in the serum (**A**), cortex (**B**), hippocampus (**C**), and cerebellum (**D**). Tables display statistical analysis of immune challenge (Imm), adjuvant-only (Adj) and saline (Sal) conditions with representative colors: **red**, p<0.05; **light red**, 0.05<*p*<0.1; **dark blue**, p<0.05; **light blue**, 0.05<*p*<0.1. Red coloring overall corresponds initial treatment conditions over the second in the row; e.g. a red cell in Imm/Sal row is interpreted as significant increase in Imm compared to Sal for that analyte, blue cells are the inverse relationship and would represent a decrease in Imm compared to Sal. A value of b.d. is indicative of cytokine/chemokine values below the level of assay detection. (JPG 1756 kb)

## Data Availability

The datasets included in this study are available from the corresponding author upon reasonable request.

## Abbreviations

BN: Brown Norway
CNS: Central nervous system
GFAP: Glial fibrillary acidic protein
HPA: Hypothalamus pituitary adrenal
IBA-1: Ionized calcium-binding adaptor molecule 1
IgG: Immunoglobulin
IHC: Immunohistochemistry
iNOS: Inducible nitric oxide synthase
LPS: Lipopolysaccharide
MIA: Maternal immune activation
Th: T helper
