# Supplementary figures and images for: Acute peripheral immune activation alters cytokine expression and glial activation in the early postnatal rat brain
Source: J Neuroinflammation. 2019 Oct 31;16:200. doi: 10.1186/s12974-019-1569-2 (PMC6822372; doi:10.1186/s12974-019-1569-2)

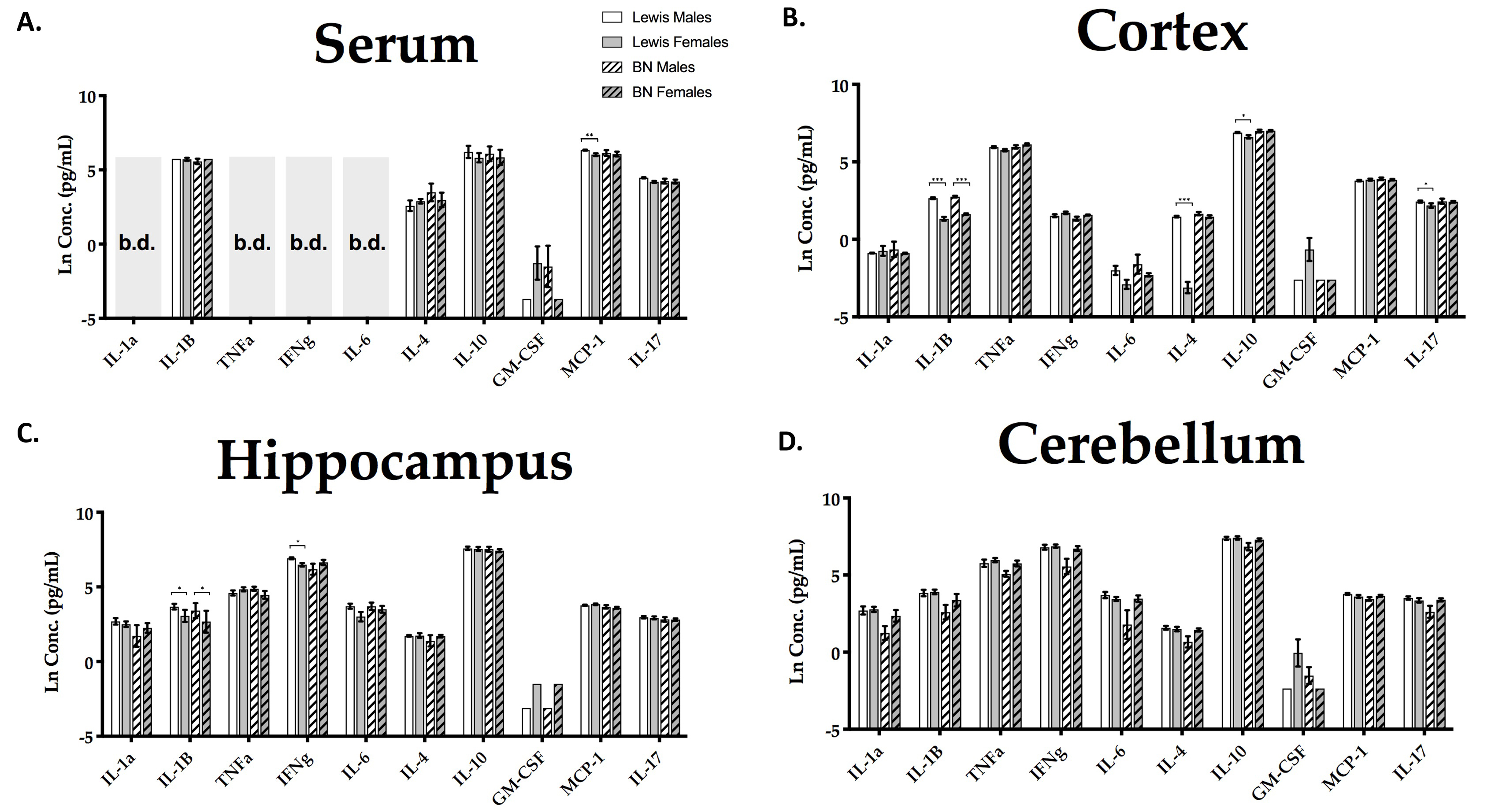

Supplement: Supplementary file 1 — Baseline cytokine results. Analysis of cytokine levels in response to saline-only conditions. Results represent cytokine concentrations in the serum (A), cortex (B), hippocampus (C), and cerebellum (D) of male and female Lewis (N=15; 7M, 8F) and BN (N=15; 6M, 9F) rats. Data represent mean +/- SEM, collapsed between day of collection. Value b.d. represents analytes where >50% of samples were below the level of detection and excluded from analysis; *p<0.005, **p<0.01, ***p<0.001. (JPG 1626 kb) [file 12974_2019_1569_MOESM1_ESM.jpg]

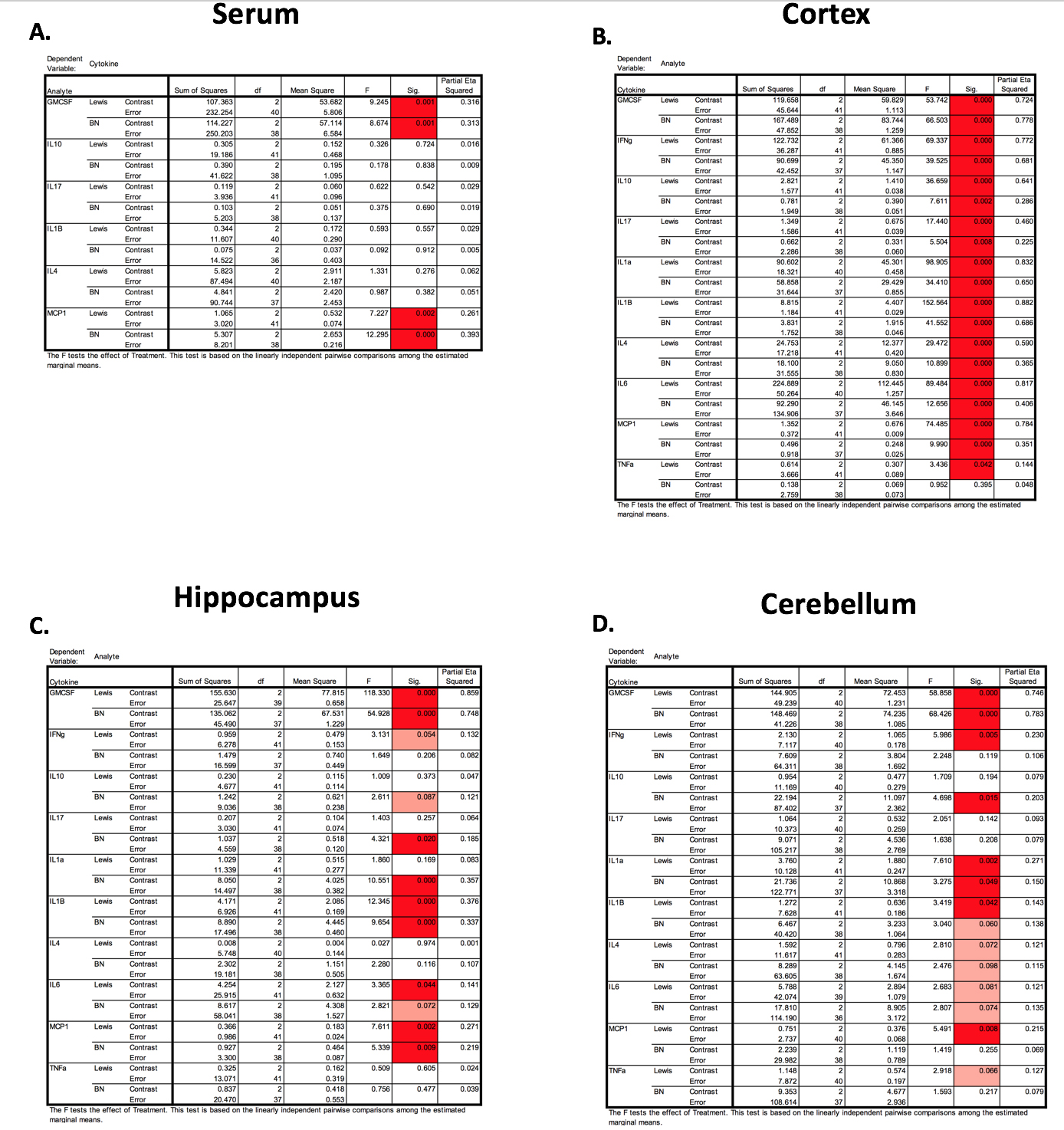

Supplement: Supplementary file 2 — Main effect of treatment on cytokine levels. Results of ANOVA analyses considering a main effect of treatment on cytokine and chemokine levels in the serum (A), cortex (B), hippocampus (C), and cerebellum (D) of Lewis and BN rats. Red coloring denotes a significant finding (p<0.05), while pink coloring represents a trending result (0.05<p<1). (JPG 1038 kb) [file 12974_2019_1569_MOESM2_ESM.jpg]

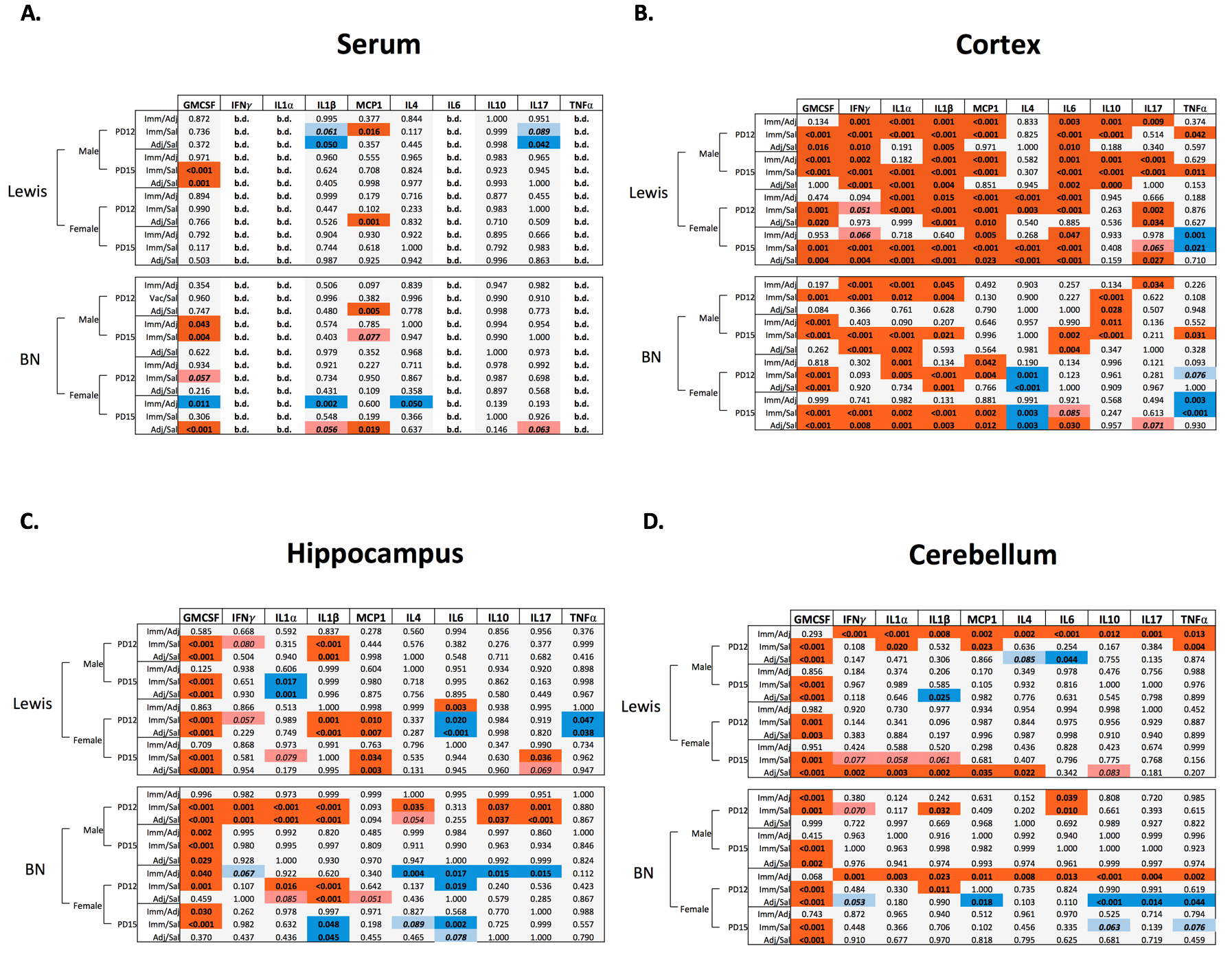

Supplement: Supplementary file 3 — Total cytokine and chemokine protein analyses of serum and brain lysates. Multi-factorial ANOVA analyses were conducted; p-values displayed here reflect Sidak-adjusted values for multiple comparisons. Tabular results of comparative cytokine and chemokine analyses between treatment conditions and within day of collection, sex, and strain; split between results in the serum (A), cortex (B), hippocampus (C), and cerebellum (D). Tables display statistical analysis of immune challenge (Imm), adjuvant-only (Adj) and saline (Sal) conditions with representative colors: red, p<0.05; light red, 0.05<p<0.1; dark blue, p<0.05; light blue, 0.05<p<0.1. Red coloring overall corresponds initial treatment conditions over the second in the row; e.g. a red cell in Imm/Sal row is interpreted as significant increase in Imm compared to Sal for that analyte, blue cells are the inverse relationship and would represent a decrease in Imm compared to Sal. A value of b.d. is indicative of cytokine/chemokine values below the level of assay detection. (JPG 1756 kb) [file 12974_2019_1569_MOESM3_ESM.jpg]
